# Fructose decreases physical activity and increases body fat without affecting hippocampal neurogenesis and learning relative to an isocaloric glucose diet

**DOI:** 10.1038/srep09589

**Published:** 2015-04-20

**Authors:** Catarina Rendeiro, Ashley M. Masnik, Jonathan G. Mun, Kristy Du, Diana Clark, Ryan N. Dilger, Anna C. Dilger, Justin S. Rhodes

**Affiliations:** 1 Beckman Institute for Advanced Science and Technology, 405 N. Mathews Ave., Urbana, IL 61801; 2 Center for Nutrition, Learning and Memory, University of Illinois at Urbana-Champaign; 3 Department of Psychology, University of Illinois at Urbana-Champaign; 4 Division of Nutritional Sciences, University of Illinois at Urbana-Champaign; 5 Department of Animal Sciences, University of Illinois at Urbana-Champaign

## Abstract

Recent evidence suggests that fructose consumption is associated with weight gain, fat deposition and impaired cognitive function. However it is unclear whether the detrimental effects are caused by fructose itself or by the concurrent increase in overall energy intake. In the present study we examine the impact of a fructose diet relative to an isocaloric glucose diet in the absence of overfeeding, using a mouse model that mimics fructose intake in the top percentile of the USA population (18% energy). Following 77 days of supplementation, changes in body weight (BW), body fat, physical activity, cognitive performance and adult hippocampal neurogenesis were assessed. Despite the fact that no differences in calorie intake were observed between groups, the fructose animals displayed significantly increased BW, liver mass and fat mass in comparison to the glucose group. This was further accompanied by a significant reduction in physical activity in the fructose animals. Conversely, no differences were detected in hippocampal neurogenesis and cognitive/motor performance as measured by object recognition, fear conditioning and rotorod tasks. The present study suggests that fructose *per se*, in the absence of excess energy intake, increases fat deposition and BW potentially by reducing physical activity, without impacting hippocampal neurogenesis or cognitive function.

The rapidly increasing rates of obesity is a major concern in the western world. Whilst there are likely numerous contributors to the epidemic, one potential harmful factor that has attracted much attention is the increasing intake of simple carbohydrates, such as fructose^1^. Analysis of the western diet has shown that fructose has increasingly supplied a greater proportion of total caloric intake in the last 40 years, with over 10% of American's daily calories derived from fructose^2^. It is further estimated that among the population ranked in the top fructose consumers, calorie intake coming solely from this simple carbohydrate can range from 15% (among male adolescents) up to 23% (top 1% of the population)^3^,^4^. Nonetheless, despite the parallel increase in obesity and levels of fructose intake, the specific contribution of this monosaccharide to overweight and obesity in the population remains debatable. In reality, both the total amount of calories consumed as well as the percentage of calories deriving from fructose are increasing concurrently in today's society. As a consequence, it is not clear whether fructose *per se* contributes to the negative metabolic outcomes described in the absence of overfeeding.

In the recent years, there has been a considerable volume of research in both animals and humans dedicated to clarify the link between dietary fructose and health risk markers (reviewed in Refs. 5–7). Overall effects on body weight (BW)^8^,^9^, fat deposition in adipose tissue and liver^10^,^11^,^12^, dyslipidemia^13^,^14^,^15^ and insulin resistance^10^,^12^ have been reported in human intervention trials as the main metabolic disruptions caused by fructose consumption. Fructose, unlike glucose, is primarily metabolized in the liver and is believed to favor *de novo* lipogenesis and result in hyperlipidemia^16^,^17^. Specifically, the fact that fructose bypasses the rate limiting step in glycolysis (catalyzed by phosphofructokinase) means that when significant amounts of fructose are consumed, fructose carbon continues to enter the glycolytic pathway, facilitating low density lipoprotein and triglyceride production^18^. As such, both *de novo* lipogenesis and changes in lipid profiles seem to be closely associated with the adverse metabolic effects of fructose, possibly contributing to weight gain and insulin resistance (reviewed in Refs. 7, 19). Despite the consensus on the mechanisms underlying fructose metabolism, the scientific evidence on the metabolic outcomes of fructose consumption remains equivocal. In fact, several recent meta-analyses of human isocaloric-controlled feeding trials seem to suggest that in the absence of excess energy coming from fructose there is no significant overall effect of this monosaccharide on BW^20^,^21^,^22^, blood lipids^23^,^24^, blood pressure^25^ and liver fat^26^. Altogether, this strongly suggests that the adverse effects observed are likely to be due to excess calorie intake rather than to the fructose itself. However, it is important to take into consideration that most of the human data relies on short-term interventions, so it is fairly difficult to draw conclusions regarding the long-term effects of fructose consumption. Interestingly, the greatest percentage of research in fructose comes from mechanistic animal studies which overall report pronounced metabolic disruptions, mainly related to increases in *de novo* lipogenesis, dysregulation of lipid profiles, insulin resistance and BW gain^5^,^7^,^13^,^19^,^27^. However, most animal interventions were not designed to control for caloric intake, and consistently used unrealistically high doses of fructose (ranging from 30% up to 60% of energy derived from fructose) in their study designs^28^. This drastically limits the significance of such studies to the human population and fails to address relevant questions related to whether fructose *per se* drives such adverse metabolic outcomes.

Most recently, there has been a growing interest in the potential secondary effects of fructose intake in the central nervous system (CNS)^29^,^30^, with reports showing that metabolic disruptions such as diminished insulin responses within the peripheral tissues can affect insulin signaling in the brain and may contribute to synaptic plasticity and cognitive impairments^27^,^31^,^32^. Several recent studies suggest that intake of fructose, at doses ranging from 35% to 60% of energy coming from this simple carbohydrate, induce impairments in spatial memory and learning^33^,^34^,^35^ in rodents, with the main mechanisms reported being related to alterations in hippocampal insulin signaling, insulin-like growth factor-1 (IGF-1) and brain derived neurotrophic factor (BDNF) levels^34^, reductions in hippocampal neurogenesis^36^ and increases in neuroinflammatory markers^35^,^36^. However, similarly to previous animal interventions, the interpretation of the reported negative effects of fructose in the CNS is difficult due to the high doses of fructose administered and the use of non isocaloric controls. As such, energy balance and excess calories^27^ remain important confounders in assessing the role of fructose in both metabolic health and cognitive function.

The present investigation specifically examines the impact of a long-term intervention (11 weeks) with fructose in relation to an isocaloric glucose diet at levels of energy derived from simple carbohydrates that reflect the intake of fructose top consumers in the USA population (18% energy provided from simple carbohydrates) on both the periphery and the CNS. Specifically we examined changes in BW, body fat, levels of physical activity, motor performance, learning and memory, and adult hippocampal cell genesis as a marker for neuronal plasticity.

## Methods

All experimental procedures involving animals were conducted in accordance to the regulations and guidelines set forth by the United States Department of Agriculture (USDA) and the Association for Assessment and Accreditation of Laboratory Animal Care (AAALAC). All the experimental procedures were approved by the Institutional Animal Care and Use Committee (IACUC) of the University of Illinois.

### Animals

Male C57BL/6J mice (N = 24) at 22 days of age were obtained from The Jackson Laboratory (Bar Harbor, ME). Upon arrival, mice were group-housed with four animals per polycarbonate shoebox-style cage (29 × 19 × 13 cm) and acclimatized to a reversed 12 h photoperiod (lights on at 1000 and off at 2000) in a temperature-controlled (21 ± 1°C) room for one week. During this acclimation period, animals were provided *ad libitum* access to AIN-93G purified rodent diet (Teklad 94045; Harlan Laboratories, Indianapolis, IN) and fresh water. Cages were furnished with 1/4″ corncob bedding (Teklad 7097; Harlan Laboratories), cotton squares, and cardboard tubes for nesting and environmental enrichment.

### Experimental Design

Two isocaloric treatment diets containing fructose or glucose at 18% of total metabolizable energy were formulated based on the AIN-93G purified rodent diet^37^ and manufactured by Custom Animal Diets (Bangor, PA). Both diets replaced all sucrose and a fraction of cornstarch with either glucose or fructose. Analysis of the two diets for proximate composition was performed to validate that both diets were indeed equivalent in metabolizable energy content as based on their respective crude nutrient profiles (Table 1).

One week after arrival, animals were singly housed and randomly assigned to receive either fructose or glucose treatment diets (n = 12/group) for a total of 11 weeks (77 days) in order to assess the long-term effects of the interventional diets. Food intake and BW were measured daily at the end of the light cycle for 77 days. On days 31–40, animals received daily i.p. injections of 50 mg/kg bromodeoxyuridine (BrdU) to label dividing cells. On days 61–77, mice were evaluated for behavioral performance in a battery of tests. First, animals were placed into custom-made home cages for continuous video tracking of home cage activity over 5 days. The animals were then returned to their standard cages for 2 days where they remained undisturbed except for daily weighing of food and BW. Next, animals were evaluated for novel object recognition (3 days), then rotarod (3 days). The following 2 days animals remained undisturbed. Finally, animals were tested for contextual fear conditioning (2 days). Each of these tasks is described in more detail below. On day 78, mice were anesthetized with sodium pentobarbital (100 mg/kg, i.p.), and then transcardially perfused with 0.9% saline followed by 4% paraformaldehyde in phosphate buffer solution (PBS; 0.287% sodium phosphate monobasic anhydrous, 1.102% sodium phosphate dibasic anhydrous, 0.9% sodium chloride in water). The liver, both right and left inguinal and subscapular fat pads and spleen were immediately dissected and weighed. The brain was also immediately dissected, post-fixed in 4% paraformaldehyde overnight at 4°C then transferred to 30% sucrose (w/v) in PBS at 4°C until sectioning.

### Home cage activity testing

Animals were placed into clear acrylic arenas (68 × 36 × 16 cm) that were divided into four individual cages (34 × 18 × 16 cm) that allowed for physical contact and social interaction through wire grating, as previously described^38^. Cages were furnished with corncob bedding. Food and water were delivered from the side so as not to interfere with video tracking. Ceiling-mounted video cameras interfaced to computers running TopScan video tracking software (Clever Sys Inc., Reston, VA) were used to continuously measure distance traveled in the cage over 5 days during both the light and dark cycle. Red light not visible to the mice was used for video tracking during the dark cycle. As demonstrated in our previous experiments^38^, it takes a minimum of 3 days for animals to habituate and acclimatize to the cages. As such, activity in days 4 and 5 (in a 5 day protocol) were found to be reliable and reproducible measures of locomotor activity. Days 4 and 5 were chosen *a priori* to data collection in the present experiment as reliable measures of locomotor activity.

### Energetic cost of home cage activity

The energetic cost of voluntary home cage physical activity in our study was estimated following Koteja et al.^39^. First, we estimated the macronutrient concentrations available to the animal for generation of usable energy (i.e., metabolizable energy), taking into consideration: a) food disappearance, b) the energy excreted in the form of feces (estimated at 22% of total food intake) and urine (estimated at 3% of total food intake) as previously shown^39^, and also c) food wastage due to spillage in the home cage (estimated at 10% of total food intake), as there is considerable evidence that food wasting is a significant source of error when estimating food intake^40^,^41^. Finally, a multiple regression was conducted predicting metabolizable energy intake over the 5 day home cage activity test (measured in grams of food) as a function of BW and total distance traveled (in km).

Based on the energy expenditure values, it was also possible to make an educated guess of the consequence of the differential energy expenditure on BW between the groups at the end of the 11 week intervention using an empirical energy balance technique as shown previously^42^ *[Energy intake – Energy expenditure = Energy storage _(fat mass+ fat free mass)_]*. In the energy balance, we take into consideration the energy density (ρ _FM_; ρ _FFM_), metabolic rates (γ _FM_; γ _FFM_) and deposition cost (γ _FM_; η _FFM_) for both fat mass (FM) and fat free mass (FFM)^43^.

### Learning, memory and motor performance

#### Novel Object Recognition

On day 1, mice were placed in the testing arena (50 × 38 × 20 cm) for 10 min to habituate to the environment. The following day, two identical objects were placed into the arena. Mice were allowed to explore the objects for thirty seconds and then immediately returned to their home cage. On day 3 animals were again placed into the arena, this time with one familiar object (explored the previous day) along with a novel object that the animal had never explored. The mice were allowed to explore the arena and the objects for a total of 5 min during which time TopScan measured the duration spent sniffing each object (using the sniffing module in Topscan). The automated measure obtained with Topscan sniffing module was validated against manual recording in previous pilot studies, it specifically demonstrated that the sniffing module reproduces accurately manual recording, providing a validated objective measurement. Two sets of objects were used, and animals were counterbalanced with respect to which object was familiar and novel between treatment groups. A discrimination index was calculated as the percent time sniffing the novel object divided by the percent time spent sniffing both objects on the test day using (day 3).

#### Rotarod

Following Clark et al.^44^, mice were placed on a stationary rotarod (AccuRotor Rota Rod Tall Unit, 63-cm fall height, 30 mm diameter rotating dowel; Accuscan, Columbus, OH, USA) which was then accelerated at 60 rpm/min. Latency to fall was recorded. Each animal underwent four consecutive trials a day for three days.

#### Contextual Fear Conditioning

Following Clark et al.^44^, two animals from each treatment group were randomly selected to serve as controls to not receive any shocks, while the remaining animals were assigned to receive shocks. On day one, mice were placed into the fear conditioning chamber for 180 seconds. Animals in the shock group received 2 foot-shocks (0.5 mA, duration 2 s) at 120 and 150 s. Animals in the control group received no shocks. The following day, all mice were placed into the same context for 180 seconds without any shocks. Percent time spent freezing was automatically recorded by TopScan.

### Immunohistochemistry

#### Tissue Preparation

Brains were sectioned (40 μm) by cryostat and stored in 24-well plates containing cryoprotectant (30% ethylene glycol, 25% glycerin, 45% PBS; v/v) at −20°C. A one-in-six series of sections from each animal (a series of rostral to caudal sections separated by 240 μm increments) was stained in the following way. Free-floating tissue sections were washed in tissue-buffering solution (TBS; 1.3% Trizma hydrochloride, 0.19% Trizma base, 0.9% sodium chloride) and incubated for 30 min in 0.6% hydrogen peroxide in TBS. Sections were then treated with 50% deionized formamide and 2× saline-sodium citrate (SSC) buffer for 120 minutes to denature DNA. Following DNA denaturation, sections were washed twice for 15 minutes each in SSC buffer prior to 37°C incubation in 2 M hydrochloric acid for 30 minutes. Washed sections were treated at room temperature with 0.1 M boric acid in TBS (pH 8.5), then blocked with TBS-X plus (0.3% Triton-X and 3% goat serum in TBS). Primary rat anti BrdU antibody (Cat. No.OBT0030; AbD Serotec, Raleigh, NC) was added to TBS-X plus at a dilution of 1:100 and incubated for 72 hours at 4°C. Following incubation, sections were washed with TBS and blocked with TBS-X plus for 30 minutes prior to addition of biotinylated secondary goat anti rat antibody (Cat. No. BA-9400; Vector, Burlingame, CA) in TBS-X at a dilution of 1:250 for 100 minutes at room temperature. Finally, tissue was processed using the ACB system (Cat. No. PK-6100; Vector) and stained using a diaminobenzidine (DAB) kit (Cat. No. D4418; Sigma, St. Louis, MO).

#### Image Analysis

The entire granule layer (bilateral) of the dentate gyrus was systemically photographed with a Zeiss brightfield microscope, using an Axiocam-to-computer interface with a total magnification of 100× to estimate the density of BrdU-positive nuclei (or cells). Photographs were analyzed using ImageJ software by tracing the granule layer and setting a threshold to remove the entire background excluding BrdU-positive nuclei. These automated counts were compared to hand counted values by simple linear regression. The relationship had an R^2^ value of 0.9688, represented by the equation: hand count = 0.9564*(automated count) − 0.4776. Because the automated counts slightly underestimated the value of hand-counted cells, the automated counts were corrected using the linear equation. The total number of BrdU+ cells was estimated by multiplying the linear transformed value by 0.85 to account for 15% probability that an individual BrdU+ cell was located in the top plane of the section (and therefore would be represented twice in adjacent sections) and by a factor of 6 because only 1-in-6 series was measured^44^.

### Statistical Analysis

All statistical analyses were completed using SAS version 9.1 (SAS Institute, Cary, NC). A P-value less than 0.05 was considered statistically significant. Weekly averages for food intake and BW were analyzed using a repeated measures 2-way ANOVA with week as the within-subjects factor, and dietary treatment as the between-subjects factor. Body composition measures (i.e., mass of fat pads, liver, and spleen) were analyzed as raw values and expressed as a percentage of BW using two-sample unpaired t-tests assuming equal variances between groups. Distances traveled in the home cage (km/day), the discrimination index for the novel object test, and number of BrdU positive cells in the granule layer were analyzed using unpaired t-tests. The average discrimination index across all animals was compared to zero using a one-sample t-test to determine whether the animals learned the task. Latency to fall from the rotarod was analyzed using a repeated measures 2-way ANOVA with day as the within-subjects factor, and diet treatment as the between-subjects factor. Percent time freezing in the contextual fear conditioning apparatus was analyzed by 2-way ANOVA with shock group (shock or no shock) and dietary treatment as the two factors.

## Results

### Body Weight and Food Intake

#### BW

**Figure 1:**
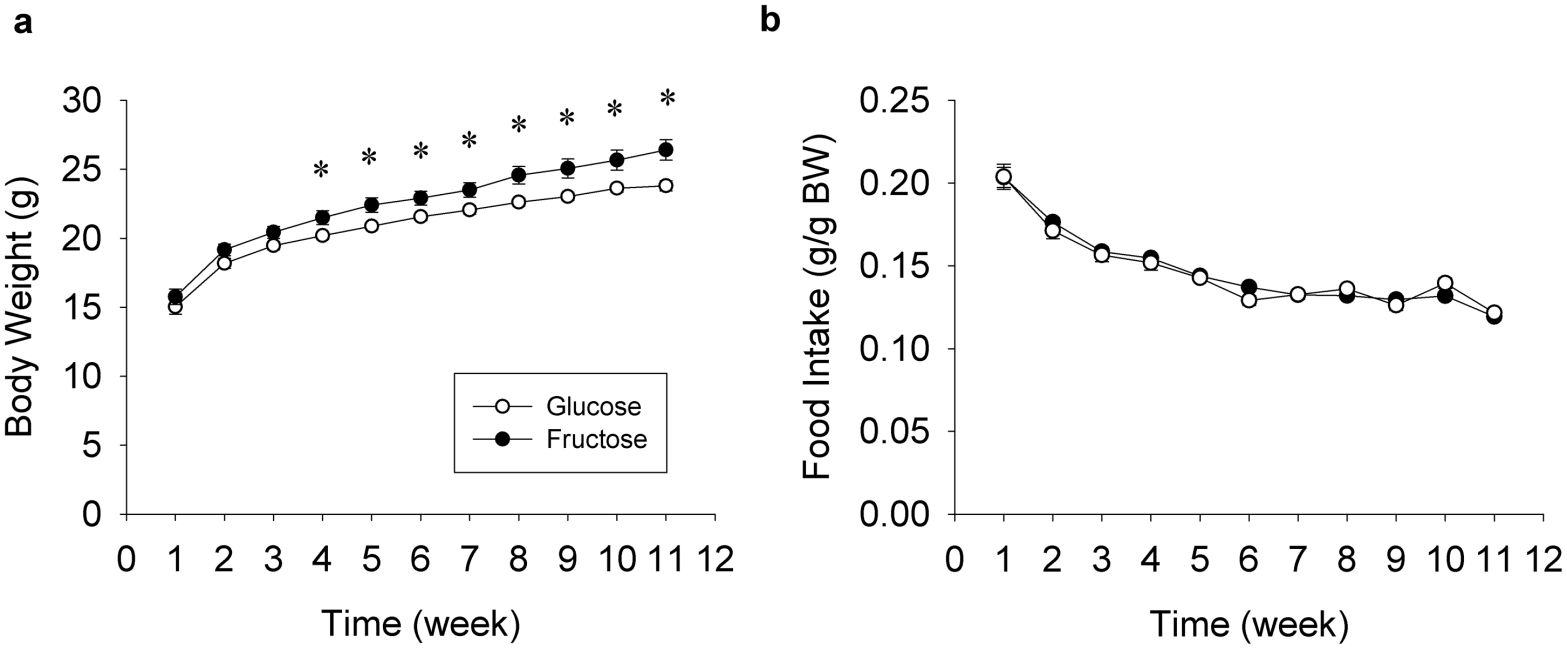
Body weight and food intake. (a) Average absolute body weight (BW) (g) per week across the duration of the study shown separately for animals fed the glucose versus fructose diets. Starting at week 4, the fructose-fed animals gained more (P < 0.05) weight than the glucose-fed animals. (b) Average consumption of food expressed relative to BW. No differences in relative food consumption were observed. Data expressed as means plus standard errors. * signifies a difference (P < 0.05) between dietary treatments on a given week of study.

At the start of the experiment, animals in glucose and fructose groups were not significantly different in BW. However, gradually over the experimental period, the fructose animals gained significantly more weight than the glucose animals (Fig. 1a). This result was supported by a significant effect of week (F_10,220_ = 443.04, P < 0.0001), treatment (F_1,22_ = 6.34, P = 0.02), and interaction between week and treatment (F_10,220_ = 4.26, P < 0.0001) in the overall repeated measures ANOVA. By week 4, average BW of animals in the fructose group was significantly greater than the glucose group (t_22_ = 2.29, P = 0.03), and the difference continued to grow during all subsequent weeks (all P < 0.01; Fig. 1a). By the end of the 11-week experiment, the fructose animals weighed on average 2.6 grams, or 11%, more than the glucose animals.

#### Food intake

Average food intake expressed as a percentage of BW was not significantly different between treatments for the entire duration of the experiment (Fig. 1b). This result was supported by a significant effect of week (F_10,220_ = 132.13, P < 0.0001), but no effect of treatment or interaction between week and treatment in the repeated measures ANOVA (Fig. 1b). Average food intake expressed as raw values not corrected for BW was significantly greater in the fructose group as compared to glucose. This was supported by a significant effect of week (F_10,220_ = 7.43, P < 0.0001), treatment (F_1,22_ = 6.77, P = 0.016), and interaction between week and treatment (F_10,220_ = 2.91, P = 0.002). By week 8, average food intake in the fructose group was significantly greater than the glucose group (t22 = 3.55, P = 0.002), and the difference remained significant in remaining weeks (all P < 0.01). By the end of the 11-week experiment, the fructose animals ate approximately 13% more food than the glucose animals uncorrected for BW differences.

### Tissue Mass

#### Spleen mass

**Figure 2:**
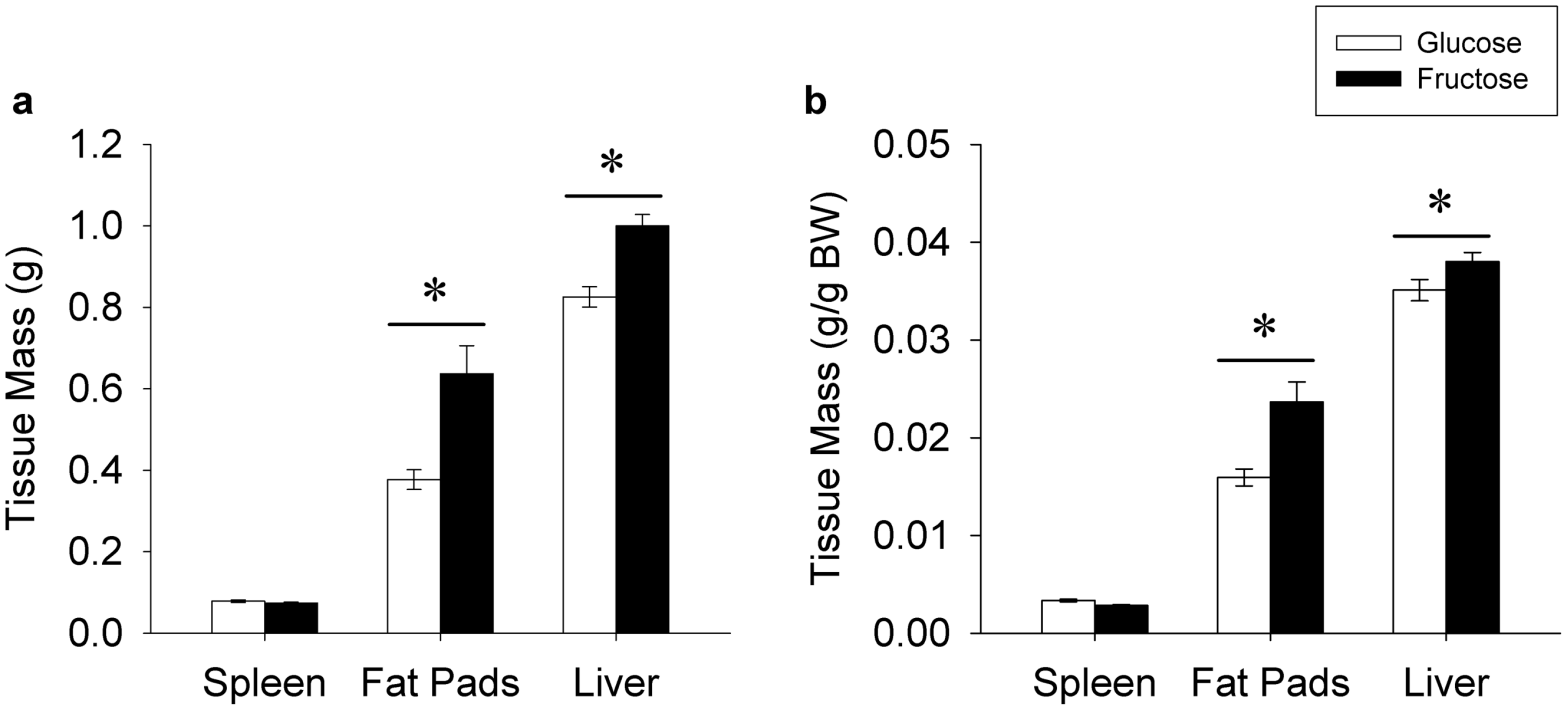
Tissue mass. (a) Average absolute mass (g) of the spleen, fat pads and liver of animals fed the glucose or fructose diets. (b) Average relative mass of the spleen, fat pads, and liver expressed per g body weight. Absolute and relative masses of the fat pads and liver were greater (P < 0.05) in fructose-fed mice compared with glucose-fed mice. Data expressed as means plus standard errors. * signifies a difference (P < 0.05) between dietary treatments.

Spleen mass was not significantly different between groups regardless of whether expressed as raw mass (Fig. 2a) or percentage of BW (Fig. 2b).

#### Fat pads

The fat pads of mice consuming the fructose diet were 69% heavier than the fat pads of animals consuming the glucose diet (t_22_ = 3.57, P = 0.002; Fig. 2a). When expressed as percentage of BW, fat pad mass remained significantly increased (t_22_ = 3.48, P = 0.002; Fig. 2b) in fructose-fed mice compared with glucose-fed mice.

#### Liver mass

The average liver mass of mice in the fructose treatment group was 20% heavier than for mice in the glucose group (t_22_ = 4.66, P < 0.0001; Fig. 2a). When expressed as percentage of BW, the difference remained significant (t_22_ = 2.02, P = 0.05; Fig. 2b).

### Home cage physical activity

**Figure 3:**
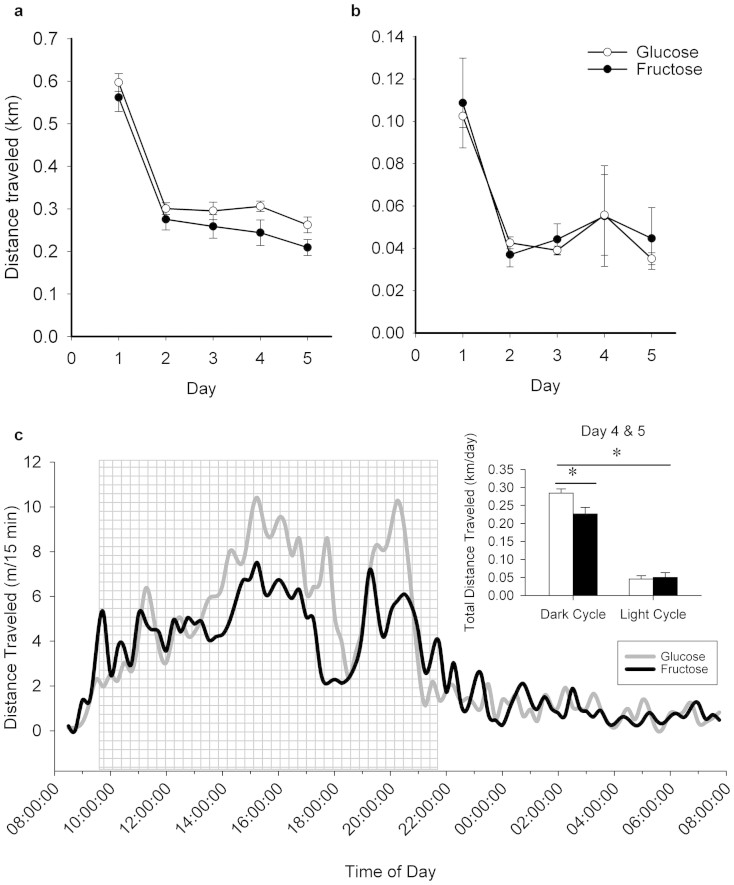
Home cage physical activity. Average distance travelled in the home cage across the 5 days of testing during the (a) dark cycle and the (b) light cycle. (c) Average distance traveled in the home cage on days 4 and 5 of the 5 day test across the light:dark cycle. The main graph shows average distance traveled in m/15 minutes over 24 hours with a smoothing function to link the 15 minute intervals. The bar graph inset at the top right shows average distance traveled (±SE) across days 4 and 5 shown separately for the dark and light cycle. Glucose-fed animals are shown separately from fructose-fed animals. Glucose-fed animals were more (P < 0.05) active than fructose-fed animals.

Home cage activity across the 5 days of testing is presented for both the dark cycle (Fig. 3a) and the light cycle (Fig. 3b). Days 4 and 5 were chosen *a priori* to data collection as reliable measures of locomotor activity based on our previous experiments demonstrating that it takes a minimum of 3 days for animals to acclimatize^38^. Specifically, during the dark cycle on days 4 and 5, mice given the fructose diet traveled significantly less (approximately 20% less) in comparison to animals fed the glucose diet (t_20_ = 2.17, P < 0.05) (Fig. 3c). No differences in cage activity were detected during the light cycle, as expected. Two animals in the glucose treatment group were excluded from data analysis due to erroneous video tracking.

### Energetic cost of home cage activity

**Figure 4:**
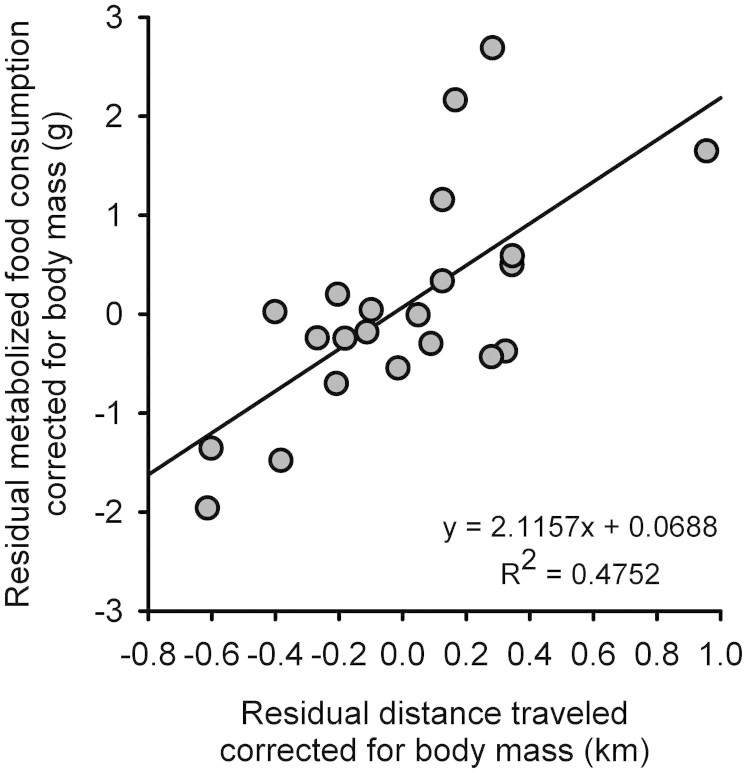
Energetic cost of physical activity. To estimate the energetic cost of physical activity we regressed estimated metabolized food intake (grams) over the 5 day home cage activity test as a function of BW (grams) and total distance traveled (km). The slope of the distance coefficient was 2.1 grams (±0.51 SE) of food per km traveled. This is represented graphically as a plot of the residuals from simple linear regressions of each variable (metabolized food intake and distance traveled) on BW. The results suggest that the energetic cost of home cage physical activity was approximately 2.1 grams of metabolized food per km traveled.

The energetic cost of home cage physical activity was estimated using a multiple regression of estimated metabolized food intake (grams) over the 5 day home cage activity test as a function of BW (grams) and total distance traveled (in km)^39^. Both BW (F_1,19_ = 5.3, P = 0.03) and distance traveled (F_1,19_ = 17.2, P = 0.0005) were significant predictors of metabolized food intake. The slope of the relationship between distance and food intake after correcting for differences in BW was 2.1 grams (±0.51 SE) of food per km, implying that mice must metabolize 2.1 grams of food (8.59 kcal) per km to support the movement over and above energy needed for basal metabolism (Fig. 4). As such, we estimate an average energy expenditure of 1.95 (±0.18 SE) kcal/day for the fructose group and of 2.44 (±0.11 SE) kcal/day for the glucose group. Based on these energy expenditure values, we estimated the interval range for the difference in total BW between the two groups to be 2.4 g – 3.9 g, the actual difference detected was 2.6 (±0.83 SE) g. The lower value in the interval assumes all extra energy in the fructose group was converted to fat mass. The upper value in the interval assumes 50% was converted to fat and 50% was converted to fat free mass. As an approximation, we also assumed that the difference in energy expenditure between the groups was observed just for 50% of the days of the intervention period, because we detected no differences in physical activity between groups for the first few days of acclimatization during the physical activity test.

### Learning, memory and motor performance

#### Novel object recognition

**Figure 5:**
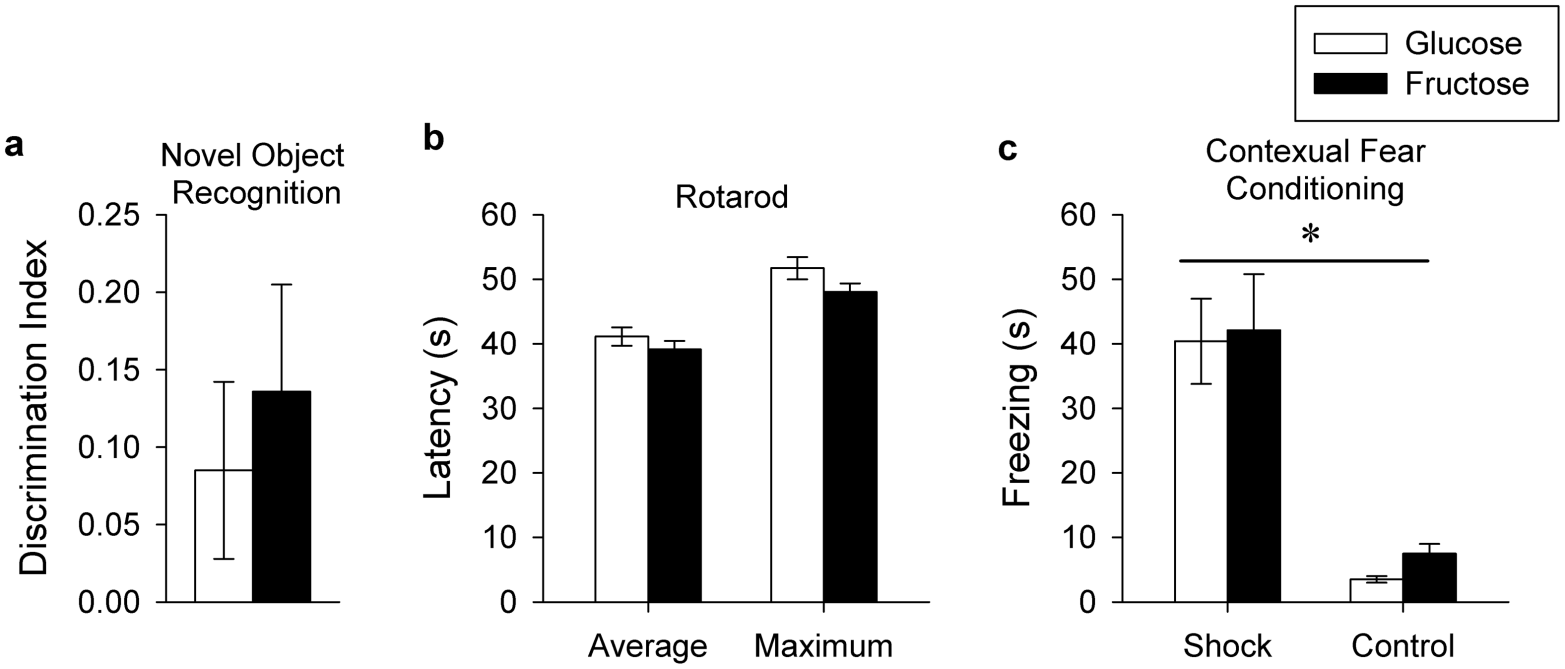
Learning, memory and motor performance. (a) Novel object recognition. Average discrimination index (±SE) expressed as the duration spent sniffing the novel object divided by the duration spent sniffing both the novel and familiar objects. No differences between dietary treatment groups were observed. (b) Rotarod performance. Average (±SE) and maximum latency (s) to fall from the accelerating rotarod shown separately for glucose and fructose groups. No differences were observed between groups. (c) Contextual fear conditioning. Average percent time spent freezing (±SE) in animals that received shocks on day 1 (shock) or no shocks on day 1 (control) shown separately for glucose-fed and fructose-fed animals. Animals learned the task as indicated by a difference (P < 0.05) between the shock and control groups, but no differences between the diet groups were observed.

The animals spent a greater (t_23_ = 2.49, P = 0.02) percentage of time sniffing the novel object as compared to the familiar object as measured by the discrimination index, however there were no differences between treatment groups (Fig. 5a).

#### Rotarod

Latency to fall from the rotarod did not differ between the fructose and glucose treatment groups on any of the days 1–3, whether expressed as average latency across the 4 trials per day or the maximum latency across the 4 trials per day (Fig. 5b).

#### Contextual fear conditioning

Animals in both groups learned the task as indicated by increased (F_1,20_ = 8.27, P = 0.0093) duration of freezing behavior in the shocked groups compared with non-shocked controls. No differences between the treatment groups were detected in either shocked or non-shocked subgroups (Fig. 5c).

### Adult Hippocampal Neurogenesis

**Figure 6:**
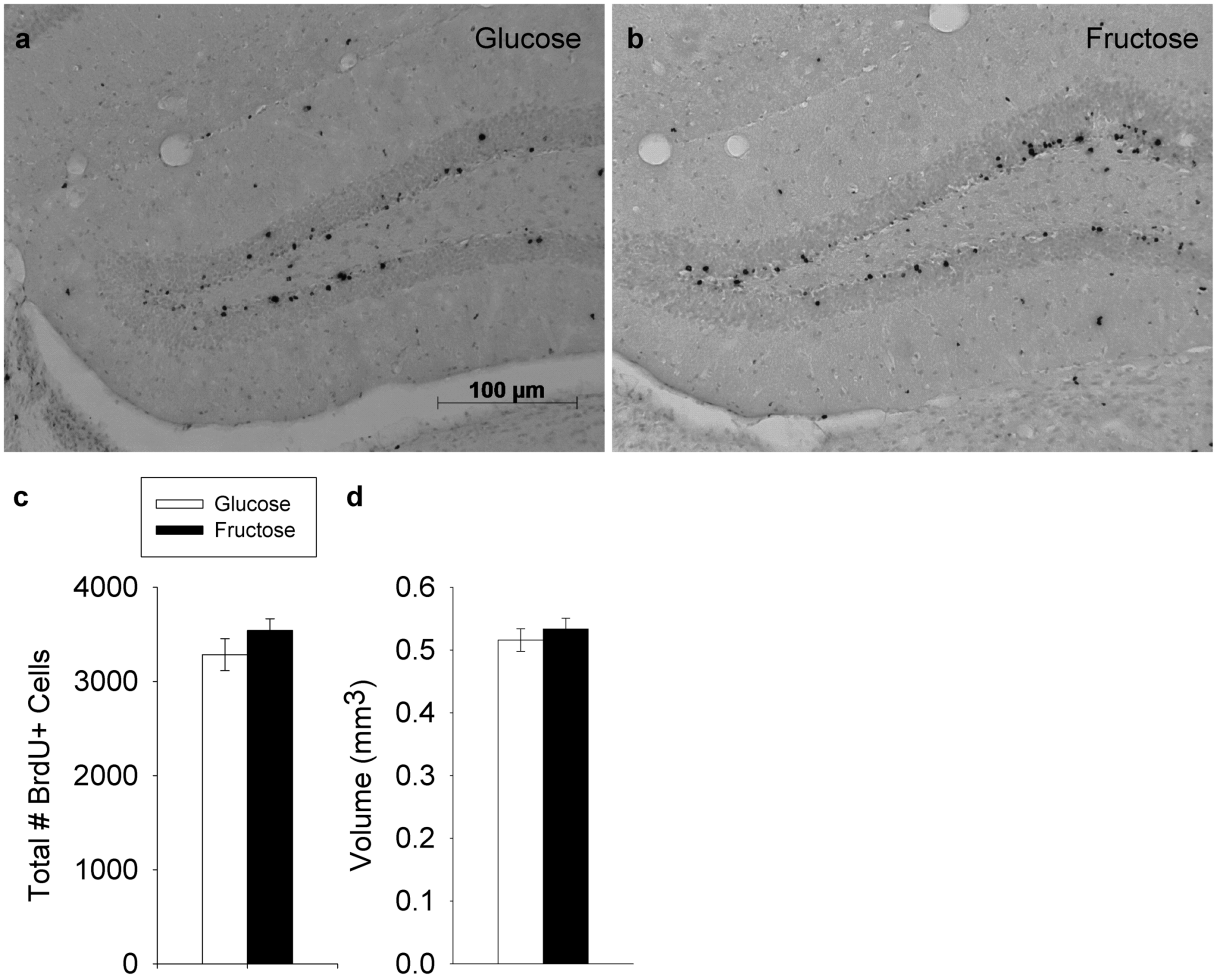
Adult hippocampal neurogenesis. (a) Representative section through the dentate gyrus of the hippocampus immunohistochemically-stained to reveal BrdU+ cells from a glucose-fed animal. (b) same as A from a fructose-fed animal. (c) Average total number of BrdU+ cells (±SE) in the granule cell layer of the dentate gyrus shown separately for animals in the glucose versus fructose groups. Previous work has established that approximately 80–90% of these cells display a mature neuronal phenotype^44^,^45^. No differences between dietary treatment groups were detected. d) Average volume of the granule cell layer of the dentate gyrus (±SE). No differences between groups were detected.

The total number of BrdU-labeled cells did not differ between dietary treatment groups. The dentate gyrus of animals fed either of the two dietary treatment maintained a comparable overall volume, as well as density of BrdU-positive cells (Fig. 6). Previous work has established that approximately 80–90% of these cells display a mature neuronal phenotype^44^,^45^.

## Discussion

Despite substantial ongoing research in both animal and humans, it has been difficult to reach a consensus regarding the roles played by added simple carbohydrates, particularly fructose, in the recent rise of obesity and related metabolic diseases^46^,^47^,^48^. Whilst there are many studies showing that consumption of fructose in comparison to other monosaccharides results in increased *de novo* lipogenesis, dyslipidemia, insulin resistance, BW^6^,^7^ and, most recently, impaired cognitive function^30^, typically such outcomes come from hypercaloric dietary interventions, with an exceedingly high percentage of energy deriving from simple carbohydrates. In the present study, we specifically examined both peripheral and central effects of glucose and fructose in young mice, when consumed as part of isocaloric diets that model the pattern of fructose consumption by adolescents in North America^2^,^49^. Furthermore, in the present study, the intake of fructose by mice was more similar to that of typical human consumption in comparison to previous studies^33^,^36^ and diets were balanced for macro and micronutrient consumption.

In comparison to calorically-equivalent consumption of diets containing glucose, prolonged consumption of diets containing fructose (11 weeks) increased BW and body fat deposition. Similarly, Bremer et al.^50^, found that healthy middle-aged rhesus macaques experienced significant increases in BW and adiposity when fed 30% daily energy intake from fructose for 1 year despite minimal changes in calorie intake (3%). These results are also similar to animal studies in which increases in fat deposition and liver mass were observed when rodents were fed fructose compared with other purified monosaccharides; however such interventions were unmatched for calorie intake^51^,^52^. Furthermore, studies in humans confirm that fructose, but not glucose (when provided as 25% of energy requirements), in the context of an energy-balanced diet increases *de novo* lipogenesis and visceral adiposity along with dyslipidemia, decreases insulin sensitivity^10^,^12^ and decreases in fat oxidation^53^. Curiously, in human studies that use isocaloric diets with subjects in energy balance (i.e., equal energy intake and expenditure), observed no overall effects of fructose in BW gain^20^,^21^. However, it is worth mentioning that such interventions in humans tend to be of very short duration (up to 4 weeks), so it is not clear whether such null effects on BW will persist in longer term interventions. In the present study, we addressed this question by using a more prolonged intervention (11 weeks) which allowed us to assess the long-term effects of fructose intake. This is also more relevant for humans since the ultimate goal is to clarify the role of fructose consumption in the obesity epidemic.

Presently, it is not clear which changes in metabolism led to the increased weight gain in the fructose relative to glucose group. Given that we observed no increases in calorie intake, we hypothesize that fructose may reduce voluntary energy expenditure in terms of physical activity. In line with this, we have detected a significant reduction (~20%) in physical activity in the fructose-fed animals in comparison to glucose. Based on the calculated energetic cost of movement of 2.1 g food/km travelled (equivalent to 8.59 kcal/km), we further estimated a decrease in energy expenditure of approximately 0.50 kcal per day for the fructose group in comparison to glucose. We also examined to what extent the difference in energy expenditure observed between the two groups could account for the difference in BW detected at the end of the intervention. Interestingly, when we calculate the appropriate balances of energy assuming that all extra energy available is converted to fat mass (as an approximation), the predicted increase in BW was approximately 2.4 g, fairly close to the observed BW gain (2.6 ± 0.83 g). On the other hand, if only 50% is converted to fat mass, the prediction of BW increase is 3.9 g. Altogether, this seems to suggest that the extra energy available was primarily converted to fat mass. Although we realize that this is just an educated guess based on a simplified law of conservation of energy, it is very reasonable to consider that the decrease in physical activity can, at least partially, account for the observed increases in BW.

In support of our observations, a recent study reported that ingestion of fructose (25% energy intake, 10 weeks) in human volunteers also resulted in reduced energy expenditure in relation to a diet with the same glucose dose^53^. Such changes in energy expenditure are likely to have important implications in regard to regulation of BW and energy balance in long-term consumption of fructose. It is realistic to consider an increase in physical activity as a way to ablate the potential negative impact of fructose consumption in BW. There is certainly evidence to suggest that, for example, exercise is able to prevent dyslipidemia in healthy subjects fed a weight-maintenance high-fructose diet (30%)^54^, which strongly suggests a protective role of physical activity in metabolic regulation.

Most recently, the potential negative effects of fructose in brain and cognitive function have been investigated, with a series of studies showing cognitive deficits in spatial memory and learning in adolescent and adult animals following access to a high fructose diet^27^,^33^,^34^,^35^. Additionally, van der Borght et al.^36^ showed that access to both fructose and sucrose, but not glucose, results in a 40% reduction in hippocampal neurogenesis. Collectively these studies seem to suggest that fructose consumption can have a considerable impact on hippocampal function and learning, which is in direct contrast with what we observed. These studies suffer from two major limitations which are common in rodent studies, specifically, they either report an unbalanced calorie intake between group treatments and/or provide a relative excess of energy from simple carbohydrates^27^,^33^,^34^,^36^,^52^,^55^, which makes interpretation of such results remarkably difficult. Typically, the unbalanced calorie intake originates from intervention ‘sugar drinks’ being added on top of an *ad libitum* diet^33^,^36^ which has been shown to result in substantial increase in overall energy intake^27^,^55^,^56^. In some studies, it is also common for fructose-fed animals to reduce their consumption of chow to compensate for the calorie intake coming from the monosaccharide solutions^35^,^50^,^52^. In these cases, there is no consumption of extra calories, yet a considerable reduction in intake of other nutrients (i.e., from chow). The altered nutrient profile is problematic as this can result in complex and widespread effects on BW and metabolic outcomes^57^. For example, in the case of a recent study reporting impaired hippocampal-dependent spatial learning in adolescent rats after 4 weeks of high fructose corn syrup consumption, there was a significant reduction in food intake in the fructose group in comparison to control (approx. 30%)^35^. This naturally results in a reduction in the intake of nutrients which is likely to contribute to the reported cognitive impairments.

In the present study, we found no significant changes in dentate gyrus neurogenesis after 11 weeks of fructose consumption along with no changes in cognitive performance. The experimental diets were matched for macro and micronutrients, and differed only in the source of simple carbohydrate. Additionally, both were modified AIN-93G diets (containing either fructose or glucose replacing sucrose and cornstarch) and contained the same caloric content as a standard AIN-93G diet (approximately 4.06 kcal/g food). Food consumption was similar to previous reports using AIN-93G in C57BL/6J mice^58^. Given that the fructose animals consumed a similar amount of food per kg BW as glucose animals throughout the experiment, the only difference in nutrient intake was the glucose/fructose manipulation. Therefore, we can conclude that when caloric consumption is equal, fructose consumption does not alter cognitive function or neurogenesis as compared with glucose consumption.

In summary, these data suggest that at lower doses (approx. 18% energy daily intake), more in line with actual levels of intake in the human population, the impact of fructose is apparent only in BW, liver mass and body fat, but not in cognitive measures or rates of neurogenesis. Further research is needed to establish whether lower doses of fructose (lower than the 18% of energy intake examined herein) would also increase BW and lipid deposition. Such dose-responses would be useful to establish what levels of dietary fructose are associated with adverse changes in both the periphery and in the CNS. Most importantly, the doses tested must be within the average and upper limits of the population consumption so mechanistic animal models can be of relevance to humans^2^,^3^,^49^. In future studies, it would also be important to compare the healthy AIN-93G diet with isocaloric fructose and glucose diets, by replacing the typical 10% sucrose with either glucose or fructose. In the present study, we could not directly compare our interventions (18% simple sugars) with the AIN-93G diet, without compromising the caloric match in sugar content among the interventions, which was the main goal of our studies. Although, we observed that at 18% of energy daily intake for 11 weeks, fructose did not affect measures of cognitive function and neurogenesis, we cannot exclude the possibility that more long-term interventions might affect brain function and cognition. Mainly, significant changes in cardiometabolic risk factors, including BW and body fat and our finding of decrease in physical activity can be associated with increased risk of cardiovascular diseases^59^ and potential detrimental effects in the CNS in the long term^60^.

## Author Contributions

C.R. wrote the introduction and discussion and analyzed and interpreted the data; A.M.M. came up with the idea for the experiment and collected most of the data; J.G.M. contributed to the experimental design, data analysis and interpretation; K.D. prepared all the figures, contributed to the experimental design, data analysis and interpretation; D.C. dissected and weighed the fat pads from the mice, contributed to the interpretation and editing of manuscript; R.N.D. analyzed the food for macronutrient content, contributed to the interpretation and editing of manuscript; A.C.D. contributed to the experimental design, specifically body composition measures, interpretation and editing of manuscript; J.S.R. contributed to experimental design, analysis interpretation and editing of manuscript.

## Figures and Tables

**Table 1:** Composition of the experimental diets

Diet	AIN-93G, Fructose	AIN-93G, Glucose
***Ingredient, g/kg (as-fed)***		
Casein	200.00	200.00
Corn Starch	311.49	311.49
Maltodextrin	132.00	132.00
** Fructose**	**186.00**	0.00
** Glucose**	0.00	**186.00**
Cellulose	50.00	50.00
Salt Mix, AIN-93G	35.00	35.00
Vitamin Mix, AIN-93	10.00	10.00
L-Cystine	3.00	3.00
Choline Bitartrate	2.50	2.50
t-BHQ	0.01	0.01
Soybean Oil	70.00	70.00
***Chemical Composition***		
Dry Matter (DM), %	86.72	88.13
Content (% of DM)		
Organic Matter, %	97.40	96.96
Crude Protein, %	19.79	19.45
Acid-Hydrolyzed Fat, %	8.20	8.28
Neutral Detergent Fiber, %	6.11	5.82
Ash, %	2.60	3.04
Digestible Carbohydrates, %^1^	63.30	63.41
***Metabolizable Energy Estimates, kcal/g***^2^		
Protein	0.792	0.778
Fat	0.738	0.745
Carbohydrate	2.532	2.536
Total	4.062	4.060
** % of kcal from monosaccharide treatment**	**18.32**	**18.33**

^1^Crude estimate of digestible carbohydrates calculated as 100 minus the sum of dietary crude protein, acid-hydrolyzed fat, neutral detergent fiber, and ash.

^2^Calulated based on dietary nutrient profile and standard physiological fuel values (i.e., ‘Atwater values’) of 4, 9, and 4 kcal/g for protein, fat, and carbohydrate, respectively.
